# Psychosocial Determinants of Attrition in a Longitudinal Study of Tobacco Use in Youth

**DOI:** 10.1100/2012/654030

**Published:** 2012-05-02

**Authors:** Ann Post, Hans Gilljam, Sven Bremberg, Maria Rosaria Galanti

**Affiliations:** ^1^Division of Social Medicine, Department of Public Health Sciences, Karolinska Institute, P.O. Box 170 70, 104 62 Stockholm, Sweden; ^2^Division of Public Health Epidemiology, Department of Public Health Sciences, Karolinska Institute, Norrbacka plan 2, 171 76 Stockholm, Sweden

## Abstract

To gain knowledge on psychosocial characteristics that predict the propensity of participation in longitudinal studies, attrition was analysed in a cohort of 3020 adolescents participating in the baseline survey of a longitudinal study with repeated followup focusing on adolescents' tobacco use. During the followup surveys, the proportion of responders was constantly at or above 90%. There were 941 adolescents (31.2%) who failed to participate in at least one of the six followup surveys. Boys had a fifty percent increased risk of nonparticipation compared with girls. Adolescents in families with experience of divorce, unemployment, and change of residence had a higher risk of nonparticipation. An increasing number of stressful life events during the previous year, uptake of tobacco use, number of friends, perceived performance at school, truancy, and alcohol use during the last term also independently associated with nonparticipation. Diverse psychosocial characteristics are independently associated with nonparticipation of youths in longitudinal studies.

## 1. Introduction

The recruitment of adolescents into longitudinal studies can be challenging, especially if repeated contacts are required. In such studies, failure of contact at any point in time entails loss of information with possible problems of validity of the results [1, 2]. Several methods for limiting or reducing the impact of attrition have been proposed: collection of data at school and tracking absentees by postal questionnaires, telephone, or home interviews [3, 4]. Payment and rewards have also been used [5]. On the other hand, the medium of data collection does not seem to have a decisive impact on response rates, as shown by studies comparing web forms versus paper-forms to collect information on alcohol and tobacco among students [6, 7]. It has been repeatedly shown, however, that youths who are prone to drop out are more likely to come from a single-parent household, to be less successful in school, and to be more often substance users [5, 8].

As far as gender is concerned, some studies have found that females are more prone to participate than males [9, 10]. Whether psychosocial characteristics affect the propensity of the adolescents to participate in longitudinal studies independently from behavioral factors is not known. In order to gain knowledge on this matter, we analyzed the information from the BROMS study (Children's Smoking and Environment in the Stockholm County), a longitudinal study of adolescents' tobacco use in the Stockholm region of Sweden. 

## 2. Materials and Methods

### 2.1. Study Population

The study sample consisted of 3020 5th grade students (1537 boys and 1483 girls), of mean age 11.6 yrs residing in the county of Stockholm in 1998. The study was initiated to assess determinants of uptake of cigarettes and snus (the traditional Swedish type of moist oral snuff). A two-step sampling was used. First, a random sample of schools in the region was selected. The guardians of the children in the consenting schools were asked to provide written permission to their children's enrolment in the cohort. Details on the study population and participation rates at recruitment both at the school and at the family level have been published previously [11].

### 2.2. Data Collection

Information from the children was collected every year (except for the first year after compulsory school), thus yielding one baseline and six followup surveys. The survey instrument was a self-completed questionnaire covering questions on health behaviors, psychosocial characteristics and experience of substance use, particularly tobacco and alcohol. At baseline and during the first four followup waves the questionnaires were completed in the classroom, sealed into an anonymous envelope and collected by the teacher. At the two remaining followup waves the questionnaires were sent to the participants' homes and returned by prepaid mail. Up to five attempts were made to reach nonresponders, twice by ordinary mail and three times by telephone, when the adolescents were given the opportunity to answer the questionnaire by phone interview. At each school survey, all participants received low-cost gifts, such as pens, while on the two surveys after compulsory school, early responders (within two weeks) were rewarded with a cinema ticket. The bulk of data collection was completed within two months during each survey. In the course of the study, tracking of participants in case of change of school or address was accomplished through the school rosters and/or the tax authority registers, using the unique national personal number as identifier.

### 2.3. Measures

#### 2.3.1. Sociodemographics

Demographic information collected at baseline included gender, age, country of birth, and parental cohabitation. Parental education was based on the highest number of school years attended by either parent at the time of the baseline survey. This was categorized as compulsory education (≤9 yrs), senior high school education (10–12 yrs), and college level education (>12 yrs).

#### 2.3.2. Psychosocial Measures

 Some selected psychosocial characteristics were collected at baseline and in grade 6, 7, and 8. Stressful events during the past year included change of residence, change of school, parental divorce, parental unemployment, and death of kindred. Besides considering each event *per se*, a categorical variable was created cumulating the total number of reported events (0, 1, 2, and above). The number of friends met regularly every week in leisure time was categorized in 0 (none), 1–4, and 5 or more. The question whether the adolescents found it easy to confide in their mother, father, or other adults was recoded into a dichotomous variable as “Confidence in any adult” (for answers: Very easy/Easy) and “No confidence in any adult” (for the answers: Difficult/Very difficult/Adult not available in every option). School truancy during the last term was categorized as 0, 1–3, ≥4 days. The judgment of own school proficiency compared to classmates was measured by the alternatives “Very good”, “Good”, “Average”, and “Below average”.

#### 2.3.3. Tobacco Use and Alcohol Consumption

 Tobacco use ever in life at the baseline survey and followup was categorized as positive answer to the question “Did you ever try smoking a cigarette, even a single puff?” and “Did you ever try snus?” Alcohol drinking was first investigated at the 7th grade survey (13 years of age) through the following questions” Did you drink any beer, wine or spirits during last school term?” with response alternatives: Never used; No; Yes, once; Yes, more than once. Intoxication drinking was assessed by the question “Have you ever consumed so much alcohol that you got drunk?” (Yes/No).

### 2.4. Outcome

At any followup survey, available (contactable) cohort members were those adolescents who took part in the baseline study, were still alive, and did not explicitly refuse continued participation prior to the date of data collection. On the basis of the response history at the end of the data collection period, we defined the available members as “Responders” if they had a completed or partially completed questionnaire responded by mail or by phone. Adolescents who had not filled in or returned the questionnaire or completed a phone interview were identified as “Non responders.” Finally, we labeled as “Dropouts” subjects who either communicated to the study team that they were not willing to further participate in the study or died during followup.

For the purpose of this study, a dichotomous outcome was considered, where nonparticipation was defined as failure to respond to any survey after baseline.

### 2.5. Statistical Analysis

Data analyses were conducted using SPSS 10.0.5 for Windows (SPSS Inc., Chicago, IL). In univariate analyses, the proportion of nonresponders in categories of the predictors was compared with the expected proportions by means of the chi-square statistic. The level for the statistical significance was conventionally set at 95% (*P* < 0.05). Predictors that were found significantly associated to non-participation in the univariate analysis were then analysed in multivariable regression models using ordinary logistic regression. Odds ratios (ORs) of nonparticipation and their corresponding 95% confidence intervals (CI) were used as measure of association and precision, respectively.

## 3. Results

Among the 3020 youths participating in the baseline survey and constituting the study sample, 2397 (79.4%) had never tried cigarettes or snus at baseline. Ever smoking was reported by 556 (18.5%) and ever snus use by 167 (5.5%) of the subjects. Current tobacco use (at least monthly) was reported by 12 subjects (0.4%). The proportion of children with at least one parent with college education was higher than the average of the regional population 49.6 percent against an average of 39.5 percent of comparable age group for the whole Stockholm County.

During the followup surveys 1 through 5, the proportion of responders was constantly at or above 90% (range 90.1–96.2%) and slightly declined only at the end of followup (Table 1).

During the study period, 181 subjects (6.0%) of those initially recruited dropped out of the study permanently, that is, either refused continued participation or died (Table 1). The dropout rates fluctuated between 0.3% and 2.0%, of the eligible, without clear trends over time. All in all, 941 adolescents (31.2%) failed to participate in one or more of the followup surveys.

In univariate analyses, most psychosocial characteristics measured at baseline were significantly associated with nonresponse any time during the study (Table 2).

Being born outside Sweden, not living together with both legal parents, several stressful life events during the year preceding the baseline survey corresponded to a higher proportion of nonparticipating subjects. Subjects reporting no friends or more than 4 friends with whom they spent their leisure time had lower participation than those reporting a group of 1–4 friends. Ever smokers at baseline, but not ever snus users, had lower participating rates than never users of tobacco. Analyses of the same predictors measured during followup resulted in very similar findings.

Some indicators of problem behaviors or psychosocial distress during followup also predicted nonparticipation in subsequent surveys. This was the case for recent alcohol drinking and intoxication drinking, smoking and snus use, school truancy, and perceived poor academic performance (Table 3).

On the other hand, access to supporting adults did not predict participation. The results described above were substantially unchanged when the analysis was restricted to the two final waves among responders in grade nine, the last grade of compulsory school. Most associations remained statistically significant in multivariate regression models including all predictors that were associated to nonparticipation in univariate analysis. Significant predictors of non-participation measured at baseline were male gender, being born outside Sweden, family circumstances and having initiated smoking (Table 4). 

On the other hand, own perception of school performance and alcohol use during last term, including intoxication drinking, no longer predicted participation after adjustment for other factors (Table 5). 

## 4. Discussion 

In this cohort study with low attrition, only 181 adolescents (6%) dropped out permanently during the six years followup period, while occasional nonparticipation slowly rose in the course of time from 3% to 12%. Consistent with previous research, females were more prone to participate than males [9, 10]. We found that many psychosocial traits and behavioural factors beside tobacco use were significant predictors of adolescents' participation. Although the choice of these predictors was somewhat arbitrary, all of them indicated either an unstable family environment or behavioural problems, such as school truancy, low school performance or substance use. An increasing number of stressful events, most of them connected with family disruption, change of residence, and/or of school, occurring in the previous year, was associated to nonresponse in a dose-response fashion. Noteworthy, the correlation of the predictors under study with nonparticipation was evident both at baseline and throughout the study period, thus indicating that the association was not dependent on the subjects' age. An interesting observation was that both the absence of close friends with whom to spend the leisure time and a high number of friends (more than 4) predicted non response, compared to having a group of 1–4 friends. These findings provide support to poorer treatment retention and followup detected in studies analysing internalizing-externalizing behaviour in youth [12, 13]. 

In multivariate analyses, we found that psychosocial distress and the uptake of tobacco use were independent predictors of nonparticipation at followup. Previous research on determinants of nonparticipation has focussed on selective loss of tobacco users, to conclude that the external validity of most surveys may be compromised by this selection [14]. However, there are longitudinal studies showing no relation between demographic or psychosocial variables, including smoking experience, and retention of the participants [15]. 

Our results show that tobacco use is neither the only nor the strongest predictor of nonparticipation. Furthermore, characteristics indicating social vulnerability were linked to adolescents' nonparticipation despite the initial selection, because children were recruited mostly from families with high social status [11]. 

### 4.1. Study Limitations and Strengths 

Although the present study did not seek to specify all psychosocial factors associated with attrition of adolescents in longitudinal studies, it may serve as a starting point identifying variables predicting retention. Strength of the findings is the repeated measurements of the psychosocial determinants during the study period. 

Our findings are in general agreement with previous observations that factors facilitating or hindering adolescent participation in studies fall into four classes: demographics, individual variables, family characteristics, and logistical factors, such as frequent changes of residence [5]. Several studies have investigated and recommended tracking and followup methods to minimize attrition [2, 3, 11, 16]. Studies of children and adolescents must develop and implement methods to increase participation of vulnerable subgroups, such as those living in conditions of psychosocial distress. Additional variables may be warranted to identify these groups and possibly increase participation rates. 

In conclusion, we suggest that baseline psychosocial characteristics of a recruited sample can be used to identify cues for an efficient followup, that is, to avoid loss of individuals of particular interest for the accuracy and validity of the observations to be conducted. 

## Figures and Tables

**Table 1:** Responders, nonresponders, and dropouts at each followup survey, The BROMS Cohort Study, 1998–2005.

	Followup survey					
	1	2	3	4	5	6
Available cohort* members	3020	3011	2976	2952	2910	2852
Responders	2904	2882	2809	2689	2621	2489
(%)	(96.2)	(95.7)	(94.4)	(91.1)	(90.1)	(87.3)
Non responders	107	94	143	221	231	350
(%)	(3.5)	(3.1)	(4.8)	(7.5)	(7.9)	(12.2)
Drop-outs	9	35	24	42	58	13
(%)	(0.3)	(1.2)	(0.8)	(1.4)	(2.0)	(0.5)

*Available cohort members are students who had not left permanently the cohort at the time of the survey.

**Table 2:** Behavioural and psychosocial characteristics at baseline as predictors of non-participation in any followup survey.

	*n*	Non responder or dropout %	*χ* ^2^	*P*
Gender				
Boys	1537	36.1	35.76	<0.000
Girls	1483	26.0		

Born in Sweden				
Yes	2817	30.4	10.51	0.001
No	195	41.5		

Mother and father living together				
Yes	2196	28.2	33.34	<0.000
No	805	39.3		

Change of residence				
No	2479	29.5	19.48	<0.000
Yes	408	40.4		

Change of school				
No	2681	30.2	9.92	0.002
Yes	184	41.3		

Parental divorce				
No	2684	30.1	17.46	<0.000
Yes	175	45.1		

Death of kindred				
No	2117	30.4	0.74	0.390
Yes	776	32.1		

Parental unemployment				
No	2607	30.0	9.88	0.002
Yes	244	39.8		

Number of stressful events last year*				
0	1698	28.3		<0.000
1	878	33.5	19.91	
>1	390	39.0		

Number of friends in leisure time				
0	20	55.0		<0.000
1–4	1818	28.4	18.97	
>4	1150	34.9		

Ever smoker				
No	2455	28.9	29.98	<0.000
Yes	556	40.8		

Ever snus user				
No	2846	30.8	1.50	0.221
Yes	167	35.3		

Ever tobacco user				
No	2397	28.7	31.23	<0.000
Yes	609	40.4		

*Includes change of residence, change of school, parental divorce, death of kindred, and parental unemployment.

**Table 3:** Behavioural and psychosocial characteristics at followup as predictors of subsequent non-participation.

	Grade when measured	*n*	Non responderor dropout (%)	*χ* ^2^	*P*
Any adult to confide in	6				
Yes		2777	28.4	0.29	0.591
No		89	25.8		

Ever smoker	7				
No		1530	19.6	67.65	<0.000
Yes		1342	33.1		

Ever snus user	7				
No		2289	22.4	72.63	<0.000
Yes		587	39.7		

Any alcohol use during latest term	7				
No		1756	22.9	19.27	<0.000
Yes		1100	30.3		

Intoxication drinking latest term	7				
No		962	27.7	7.32	0.007
Yes		477	34.6		

Ever smoker	8				
No		1197	16.0	46.97	<0.000
Yes		1608	26.9		

Ever snus user	8				
No		1943	17.8	72.91	<0.000
Yes		861	32.3		

Truancy latest term (days)	8				
0		1980	19.0	50.00	<0.000
1–3		483	26.3		
≥4		313	35.8		

Own beliefs of teachers judgement of school proficiency compared to classmates	8				
Very good		436	20.9	30.48	<0.000
Good		1219	18.5		
Average		984	25.4		
Below average		113	37.2		

Own judgement of school proficiency compared to classmates	8				
Very good		559	24.2	28.92	<0.000
Good		1270	17.5		
Average		820	27.0		
Below average		100	25.0		

**Table 4:** Odds ratios of nonparticipation according to behavioural psychosocial characteristics at baseline.

Predictor	OR^(1)^	OR^(2)^
	(95% CI)	(95% CI)
Gender (boys versus girls)	1.6 (1.4–1.9)	1.5 (1.3–1.8)
Ever smoker at baseline (versus never)	1.7 (1.4–2.1)	1.4 (1.2–1.7)
Not born in Sweden (versus born in Sweden)	1.6 (1.2–2.2)	1.6 (1.2–2.2)
Mother and father living together at baseline (no versus yes)	1.6 (1.4–1.9)	1.5 (1.2–1.8)
Change of residence (yes versus no)	1.6 (1.3–2.0)	1.3 (1.0–1.7)
Parental unemployment (yes versus no)	1.5 (1.2–2.0)	1.4 (1.0–1.8)
Number of friends in leisure time (reference: 1–4)		
None	3.1 (1.3–7.5)	2.4 (0.9–6.6)
>4	1.3 (1.2–1.6)	1.3 (1.1–1.5)

^(1)^ Unadjusted.

^(2)^Adjusted for all other predictors in table.

**Table 5:** Odds ratios of nonparticipation according to behavioural and psychosocial characteristics at different times during followup.

Predictors/Grade	OR^(1)^	OR^(2)^
	(95% CI)	(95%CI)
Grade 7		
Gender (boys versus girls)	1.8 (1.6–2.2)	1.8 (1.4–2.3)
Ever smoker (versus never)	2.0 (1.7–2.4)	1.6 (1.2–2.1)
Ever snus user (versus never)	2.3 (1.9–2.8)	1.5 (1.2–1.9)
Any alcohol use during last term (yes versus no)	1.5 (1.2–1.7)	0.9 (0.7–1.2)
Intoxication drinking last term (yes versus no)	1.4 (1.1–1.8)	1.2 (0.9–1.5)

Grade 8		
Gender (boys versus girls)	1.9 (1.5–2.2)	1.9 (1.5–2.3)
Ever smoker (versus never)	1.9 (1.6–2.3)	1.5 (1.2–1.9)
Ever snus user (versus never)	2.2 (1.8–2.7)	1.3 (1.1–1.7)

Truancy latest term (reference: no truancy)		
1–3 days	1.5 (1.2–1.9)	1.3 (1.0–1.7)
≥4 days	2.4 (1.8–3.1)	1.9 (1.4–2.5)

Own beliefs of teacher's judgement of school proficiency compared to classmates (reference: very good)		
Good	0.9 (0.7–1.1)	1.0 (0.7–1.4)
Average	1.3 (1.0–1.7)	1.1 (0.7–1.6)
Below average	2.2 (1.4–3.5)	1.5 (0.8–2.7)

Beliefs of own school proficiency (reference: very good)		
Good	1.0 (0.6–1.6)	0.7 (0.5–1.0)
Average	0.6 (0.4–1.0)	1.1 (0.8–1.5)
Below average	1.1 (0.7–1.8)	0.7 (0.4–1.3)

^(1)^ Unadjusted.

^(2)^Adjusted for gender and all predictors in the same grade.
